# Identification of a Novel Functional Corticotropin-Releasing Hormone (CRH2) in Chickens and Its Roles in Stimulating Pituitary TSHβ Expression and ACTH Secretion

**DOI:** 10.3389/fendo.2019.00595

**Published:** 2019-08-29

**Authors:** Guixian Bu, Jie Fan, Ming Yang, Can Lv, Ying Lin, Jinxuan Li, Fengyan Meng, Xiaogang Du, Xianyin Zeng, Jiannan Zhang, Juan Li, Yajun Wang

**Affiliations:** ^1^College of Life Science, Sichuan Agricultural University, Ya'an, China; ^2^Key Laboratory of Bio-Resources and Eco-Environment of Ministry of Education, College of Life Sciences, Sichuan University, Chengdu, China

**Keywords:** chicken, CRH2, pituitary, ACTH and TSH, CRH receptor

## Abstract

Corticotropin-releasing hormone (CRH), together with its structurally and functionally related neuropeptides, constitute the CRH family and play critical roles in multiple physiological processes. Recently, a novel member of this family, namely CRH2, was identified in vertebrates, however, its functionality and physiological roles remain an open question. In this study, using chicken (c-) as the animal model, we characterized the expression and functionality of CRH2 and investigated its roles in anterior pituitary. Our results showed that (1) *cCRH2* cDNA is predicted to encode a 40-aa mature peptide, which shares a higher amino acid sequence identity to cCRH (63%) than to other CRH family peptides (23–38%); (2) Using pGL3-CRE-luciferase reporter system, we demonstrated that cCRH2 is ~15 fold more potent in activating cCRH receptor 2 (CRHR2) than cCRHR1 when expressed in CHO cells, indicating that cCRH2 is bioactive and its action is mainly mediated by CRHR2; (3) Quantitative real-time PCR revealed that c*CRH2* is widely expressed in chicken tissues including the hypothalamus and anterior pituitary, and its transcription is likely controlled by promoters near exon 1, which display strong promoter activity in cultured DF-1 and HEK293 cells; (4) In cultured chick pituitary cells, cCRH2 potently stimulates *TSH*β expression and shows a lower potency in inducing ACTH secretion, indicating that pituitary/hypothalamic CRH2 can regulate pituitary functions. Collectively, our data provides the first piece of evidence to suggest that CRH2 play roles similar, but non-identical, to those of CRH, such as its differential actions on pituitary, and this helps to elucidate the roles of CRH2 in vertebrates.

## Introduction

When animals are under stress, the hypothalamus-pituitary-adrenal (HPA) axis is activated to maintain homeostasis. Hypothalamic corticotropin-releasing hormone (CRH) plays crucial roles and act as the primary mediator in this process (1–3). CRH, a 41-amino acids (aa) neuropeptide, was first isolated from the ovine hypothalamus in 1981 (4). Since then, several structurally and functionally related peptides, including urocortin I (UCN1), urocortin II (UCN2), urocortin III (UCN3), fish urotensin I (UTS1) and amphibian sauvagine (SVG), have been identified in various vertebrate classes and categorized in the CRH family (5, 6).

CRH stimulates adrenocorticotropic hormone (ACTH) secretion and synthesis from the pituitary (1–3). Besides CRH, UCN1 is also reported to have potent ACTH-releasing activity *in vitro* (7). Moreover, *in vivo* assays showed all three UCNs could increase plasma ACTH or corticosterone level, suggesting they may be involved in the regulation of the HPA axis as well (7–9). However, UCN1 expression could not be detected in the hypothalamus and pituitary stalk (10, 11), and the selective receptors for UCN2 and UCN3 were not detected in the pituitary (12–14), hinting that the actions of UCNs might be attributed by its regulation on other hypothalamic factors (9, 15), instead of its direct effects on the pituitary. Interestingly, besides ACTH, CRH could also elevate pituitary thyroid stimulating hormone (TSH) secretion and/or expression in non-mammalian vertebrates, including birds, amphibians and reptiles (16–20). Apart from their best-recognized roles in regulating stress response, the CRH family peptides are believed to regulate emotional behavior, anxiety, growth, development, reproduction, metabolism, and immunity in vertebrates (1, 21–23).

It has been well-documented that the roles of CRH-related peptides are mediated by two G protein-coupled receptors, namely CRH receptor 1 (CRHR1) and CRH receptor 2 (CRHR2), which share ~68% amino acid identity with each other (6, 21, 23). However, these neuropeptides have distinct preference for these two receptors. For instance, CRH has a higher affinity to CRHR1 than to CRHR2 in mammals, *Xenopus* and zebrafish (24–26), but it was shown to be equipotent in activating both receptors in chickens (27). UCN1 binds to them with similar affinities, but both UCN2 and UCN3 are highly selective for CRHR2 (6, 25, 28, 29). Upon ligand binding, CRHR1 and CRHR2 activation could increase the intracellular cAMP level through activation of adenylate cyclase (AC). Moreover, they could also stimulate the PLC/IP3/Ca^2+^ and MEK/ERK signaling pathways (6).

Recently, *in silico* analyses have identified a novel CRH-like peptide (CRH2) from the genome sequence of a number of vertebrates (30, 31).This putative gene was proposed as the fifth ancient member of the CRH family, which has been likely lost in placental mammals and teleosts during evolution (5). However, this novel CRH2 gene has not been experimentally characterized and its functions and physiological roles remain largely unknown. Intriguingly, although *CRH2* has been predicted to exist in the genome of zebra finches, peregrine falcons and ducks, previous studies have failed to identify it in other birds including chickens and turkeys (5, 30). Thus, it has remained an interesting question whether this gene exists in all avian species. Therefore, using chicken as the experimental model, our present study aims to (1) identify CRH2 and characterize its functionality and tissue expression; (2) investigate the actions of CRH2 on the pituitary. Our results will be the first critical step in unveiling the functionality and physiological roles of *CRH2* in vertebrates.

## Materials and Methods

### Animals

One-week-old chicks and adult chickens were supplied by a local commercial company. Chickens were sacrificed, and tissues were collected for total RNA extraction or cell culture. All animal experiments were conducted in accordance with the Guidelines for Experimental Animals issued by the Ministry of Science and Technology of People's Republic of China. All animal experimental protocols employed in this study were approved by the Animal Ethics Committee of the College of Life Sciences (Sichuan University and Sichuan Agricultural University).

### Chemicals, Primers, Peptides, and Antibodies

All chemicals were purchased from Sigma-Aldrich (St. Louis, MO) unless stated otherwise. Primers were synthesized by Tsingke Biological Technology Co., Ltd. (Chengdu, China) and listed in Table 1. Chicken (c-) CRH (SEEPPISLDLTFHLLREVLEMARAEQL-AQQAHSNRKLMEII) and CRH2 peptides (EGKPNSLDLTFHLLREFLEMSREERLA- QKALSNKLLLQSI) with the amidated C-teminus were chemically synthesized by GL Biochem Ltd. (Shanghai, China), and reconstituted at 100 μM with Dulbecco's Modified Eagle's Medium (DMEM, Hyclone) and stored at −80°C until use. Monoclonal antibody against β-actin was purchased from Cell Signaling Technology, Inc., (Beverly, MA), while anti-ACTH antibody (ab74976) was bought from Abcam (note: the validation of antibody specificity in recognizing cACTH will be described in our coming article).

**Table 1:** Primers used in this study^a^.

**Primer name**	**Sense/antisense**	**Primer sequence (5^**′**^- to- 3^**′**^)**	**Size (bp)**	**Accession no**.
**Primers used for rapid amplification of 3′-ends (3′-RACE)**				
cCRH2-U1 cCRH2-U2 cCRH2-U3	Antisense Antisense Antisense	CCT GGA GAT GTC CCG GGA GGA GA GA GAG GAG AGA CTG GCC CAG AAG GCG CT GCC CAG AAG GCG CTC AGC AAT AAG CT		KU887752
**Primers for rapid amplification of 5′-ends (5′-RACE)**				
cCRH2-L1	Sense	GGGCTTCCTCCCCTCCGTCCGTTGGGA		KU887752
cCRH2-L2	Sense	CACTTCCCTATACTCTGCAGCAGCAG		
cCRH2-L3	Sense	AACGCCTTCTGGGCCAGTCTCTCCT		
**Primers for cloning the full-length cDNA of cCRH2**				
cCRH2-rF1 cCRH2-rR1	Sense Antisense	CGGAGCAGCGGCAGCGGTAT GTTGGGACCCCTCCCGTCACT	401	KU887752
**Primers for quantitative real-time PCR**				
*cCRH2*	Sense Antisense	CGGAGCAGCGGCAGCGGTAT CTGCAGCGGGGAGCAGCTCT	139	KU887752
*cPOMC*	Sense Antisense	CTGGGGCTGCTGCTGTGTCA GAAATGGCTCATCACGTACT	207	NM_001031098.1
*cTSHβ*	Sense Antisense	GCATCAGTTTGTGCTCCTTCA ACCTTCTCGTGAACACAGTCA	305	NM_205063
*β-actin*	Sense Antisense	CCCAGACATCAGGGTGTGATG GTTGGTGACAATACCGTGTTCAAT	123	L08165.1
**Primers for preparing the *cCRH2* promoter constructs^b^**				
−989/+170 Luc	Sense	CGGGGTACCACTGTGCACTCAAGTTCT	1159	MK550695
	Antisense	CCCAAGCTTCACGGCACCCCCGGCAT		
−518/+170 Luc	Sense	CGGGGTACCGCTCAGCTGCAGTGCTCACA	688	
	Antisense	CCCAAGCTTCACGGCACCCCCGGCAT		
−112/+170 Luc	Sense	CGGGGTACCGTCCGGTGTCACGTGAGGA	282	
	Antisense	CCCAAGCTTCACGGCACCCCCGGCAT		
−989/−519 Luc	Sense	CGGGGTACCACTGTGCACTCAAGTTCT	471	
	Antisense	CCCAAGCTTCCCCTCCCACGCTGTGCTCC		
−518/−113 Luc	Sense	CGGGGTACCGCTCAGCTGCAGTGCTCACA	406	
	Antisense	CCCAAGCTTGGAGCTGTCCGTGGTGCT		

a *All primers were synthesized by Tsingke (Chengdu, China)*.

b *Restriction sites added in 5′-end of the primers are underlined*.

### Total RNA Extraction and Reverse Transcription

Four adult chickens (2 males and 2 females) were killed and various tissues including the whole brain, spinal cord, pituitary, heart, duodenum, kidneys, liver, lung, muscle, ovary, testes, spleen, pancreas, fat, skin, telencephalon, midbrain, cerebellum, hindbrain, and hypothalamus were collected. Total RNA was isolated by RNAzol (MRC, Cincinnati, OH) according to the manufacturer's instructions and dissolved in diethylpyrocarbonate-treated H_2_O (DEPC-H_2_O). Total RNA was reverse transcribed into cDNA using Moloney murine leukemia virus (MMLV) reverse transcriptase (Takara, Dalian, China), as described in our recent study (32). In brief, oligodeoxythymide and total RNA (2 μg) were mixed in a total volume of 5 μL, incubated at 70 °C for 10 min, and cooled at 4 °C for 2 min. Then, the first strand buffer, 0.5 mM each deoxynucleotide triphosphate (dNTP) and 100 U reverse transcriptase were added into the reaction mix in a total volume of 10 μL. Reverse transcription (RT) was performed at 42 °C for 90 min. RT negative controls were performed under the same condition without the addition of reverse transcriptase (32).

### Cloning the Full-Length cDNA of cCRH2

Although *CRH2* gene was previously proposed to be lost in chickens (5, 30), it was found to be present in ducks, zebra finches and peregrine falcons, suggesting the *CRH2* might also exist in chicken genome. Based on the conserved region of duck and zebra finch *CRH2*, primers were designed to amplify *CRH2* from chicken brain using 5′-/3′-RACE PCR according to the manufacturer's instructions (Clontech, Palo Alto, CA). Based on the 5′-cDNA and 3′-cDNA ends of *cCRH2*, gene-specific primers were then designed to amplify the full-length cDNA containing the complete open reading fragment (ORF) from the adult chicken brain. The amplified PCR products were inserted into pTA2 vector (TOYOBO, Osaka, Japan) and sequenced by Tsingke.

### Sequence Alignment and Gene Synteny Analysis

Amino acid sequences for alignment analysis were either predicted from their genomic sequences, or retrieved from GenBank database. The deduced amino acid sequence of CRH2 was aligned with its orthologs from other species or paralogs from chicken using ClustalW program. Chromosomal synteny analysis was performed by comparing genome regions and searching conserved neighbor genes of *CRH2* in genome database of various species (http://www.ensembl.org).

### Quantitative Real-Time PCR (qRT-PCR)

To study the spatial expression profile of *CRH2* in chicken, its mRNA levels were detected in various adult chicken tissues using qRT-PCR. This was performed on the CFX96 Real-time PCR Detection System (Bio-Rad) in a volume of 20 μL using EvaGreen (Biotium Inc., Hayward, CA) and high-efficiency *Taq* polymerase (KOD-FX, TOYOBO, Japan), as previously described (33). In brief, the reaction was carried out under the following conditions: 2 min at 94°C for denaturation, followed by 40 cycles (98°C for 10 s, 58°C for 15 s, 68°C for 20 s) of reaction and the fluorescence signal was detected at 68°C. To confirm the specificity of PCR amplification, melting curve analysis was included at the end of the PCR reaction. The mRNA levels were calculated as the ratio to that of β*-actin* and then expressed as the fold difference compared to that of the whole brain (or telencephalon).

### Investigation of cCRH2 Actions on ACTH/TSH Expression/Secretion in Cultured Chick Pituitary Cells

Using our previously established method (33), 1-week-old chicks were sacrificed and their pituitaries were harvested. Under sterile condition, pituitaries were digested by 0.25% trypsin at 37°C for 20 min. The dispersed pituitary cells were cultured at a density of 5 × 10^5^ cells/well in the Corning CELLBIND 48-well plates with medium 199 (M199) containing 15% fetal bovine serum (Gibco) at 37°C with 5% CO_2_. After 18 h of culture, the medium was removed and the cells were treated by 120 μl M199 medium containing different concentration of peptide (cCRH2/cCRH, 0.1–10/100 nM, three replicates for each treatment) for 6 h. The conditioned medium was then harvested for measurement of ACTH and the cells were lysed for measurement of β-actin level by western blotting. Due to the small molecular weight of ACTH (39 amino acids, ~5 kDa), tricine-SDS-PAGE was used to separate proteins in the conditioned medium (34).

To examine whether cCRH2 can induce pituitary *POMC* (encoding ACTH) and *TSH*β expression, cultured pituitary cells were treated by cCRH2 peptide (1–100 nM, four replicates for each treatment) for 24 h. The total RNA was then extracted from pituitary cells and subjected to qRT-PCR assay (Primers used are listed in Table 1).

### Functional Characterization of cCRH2 in Cultured Chinese Hamster Ovary (CHO) Cells

CHO cells were cultured in Dulbecco's Modified Eagle's Medium (DMEM) supplemented with 10% (vol/vol) fetal bovine serum, 100 U/ml penicillin G, and 100 g/ml streptomycin (HyClone) in a 100 mm dish (Corning) and incubated at 37°C with 5% CO_2_. As described in our previous study (35), the functionality of cCRH2 was examined in CHO cells transiently expressing CRHR1 or CRHR2 by the pGL3-CRE-luciferase reporter system, which was utilized to monitor receptor-mediated activation of the intracellular cAMP/PKA signaling pathway. The luciferase activities in each treatment group were expressed as relative fold increase as compared with that of respective control groups (i.e., without peptide treatment).

### Identification of Promoter Region of Chicken CRH2 Gene

According to the genomic sequence of *cCRH2*, gene specific primers were designed and used to amplify its 5′-flanking regions using high-fidelity *Taq* DNA polymerase (TOYOBO). The PCR products were inserted into pGL3-Basic vector and sequenced. Then a series of plasmids containing distinct lengths of 5′-flanking region of *cCRH2* were prepared. In this study, transcription start site (TSS) on exon 1 of each gene determined by 5′-RACE was designated as “+1,” and the first nucleotide upstream of TSS was designed as “−1.” Finally, the promoter activities of these constructs were determined in cultured DF-1 and HEK293T cells using Dual-luciferase Reporter assay (Promega, Madison, MI), as described in our previous study (36). Luciferase activity of promoter constructs in cells was normalized to *Renilla* luciferase activity derived from the pRL-TK vector and then expressed as relative fold increase compared with the control group (pGL3-Basic vector).

### Data Analysis

Band intensity on Western blot was semi-quantified using the Image J software (National Institutes of Health), and the relative protein levels normalized by that of intracellular β-actin were then expressed as the fold increase compared with respective controls. The relative mRNA levels of *POMC* and *TSH*β in cultured pituitary cells were calculated as the ratios to that of β-actin and then expressed as the percentage compared to their respective controls. The data were analyzed by one-way ANOVA followed by Dunnett's test using Graphpad Prism 5 (Graphpad Software, San Diego, CA). All experiments were repeated at least two to four times to validate our results.

## Results

### Cloning the Full-Length cDNA Sequence of cCRH2

**Figure 1:**
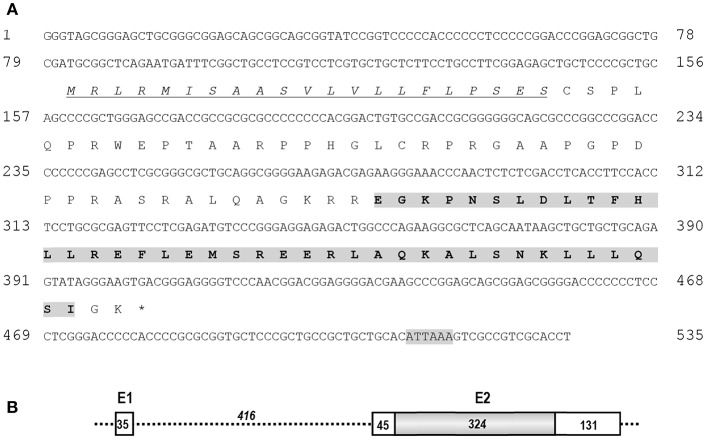
**(A)** Nucleotide and predicted amino acid sequences of *cCRH2*. The putative signal peptide was underlined and in italic. The predicted mature peptide was shaded and in bold, and the putative polyadenylation signal (ATTAAA) was shaded. **(B)** Exon (E)-intron organization of *cCRH2*. The coding region of *cCRH2* (324 bp) was intron-less and colored in gray. Numbers in the boxes indicate the size of non-coding or coding regions (shaded), and number in italic indicates the size of intron 1. The asterisk (*) indicates the stop codon.

**Figure 2:**
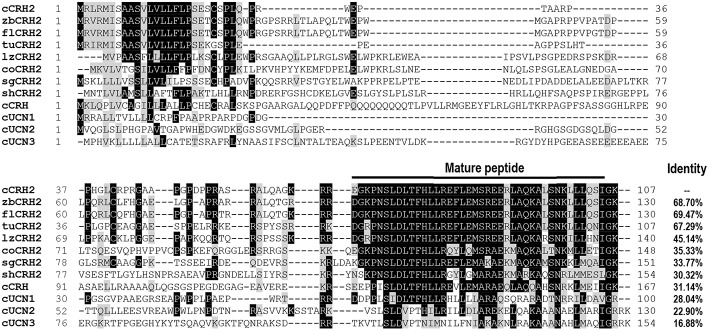
Amino acid alignment of chicken CRH2 precursor (cCRH2: KU887752) with that of zebra finches (zbCRH2), flycatchers (flCRH2), turtles (tuCRH2), lizards (lzCRH2), coelacanth (coCRH2), spotted gars (sgCRH2), elephant sharks (shCRH2), or with chicken CRH (cCRH: NP_001116503.1), UCN1 (cUCN1: XP_015140488.2), UCN2 (cUCN2: APU52336.1), and UCN3 precursor (cUCN3: AGC65587.1). The mature peptide regions were marked and dashes denote gaps in the alignment. Identical amino acid residues between cCRH2 and other precursors are shaded in black, and similar ones in gray.

Using RACE PCR, we cloned the full-length cDNA of *cCRH2* from chicken brain tissue (Accession no. KU887752). The cloned *cCRH2* is 535 bp in length and encodes a 107-aa precursor with a 21-aa signal peptide. As shown in Figure 1A, the cCRH2 precursor is predicted to encode a 40-aa mature peptide, which shares remarkable sequence identities with counterparts in other species, including zebra finch (97.5%), flycatcher (97.5%), turtle (95%), lizard (87.5%), coelacanth (73.17%), spotted gar (69.77%), and elephant shark (58.54%) (Figure 2). cCRH2 shares the highest sequence identity with cCRH (63.41%), and has comparatively lower identities to chicken UCN1 (37.50%), UCN2 (37.50%), and UCN3 (22.50%). Interestingly, the exact length of mature CRH2 differs between species, for instance, it is 41 aa in coelacanths and sharks and 43 aa in spotted gars.

Like *cCRH, cCRH2* consists of 2 exons which are separated by a 416-bp intron, and the coding region is located within exon 2 (Figure 1B).

### Synteny Analysis

**Figure 3:**
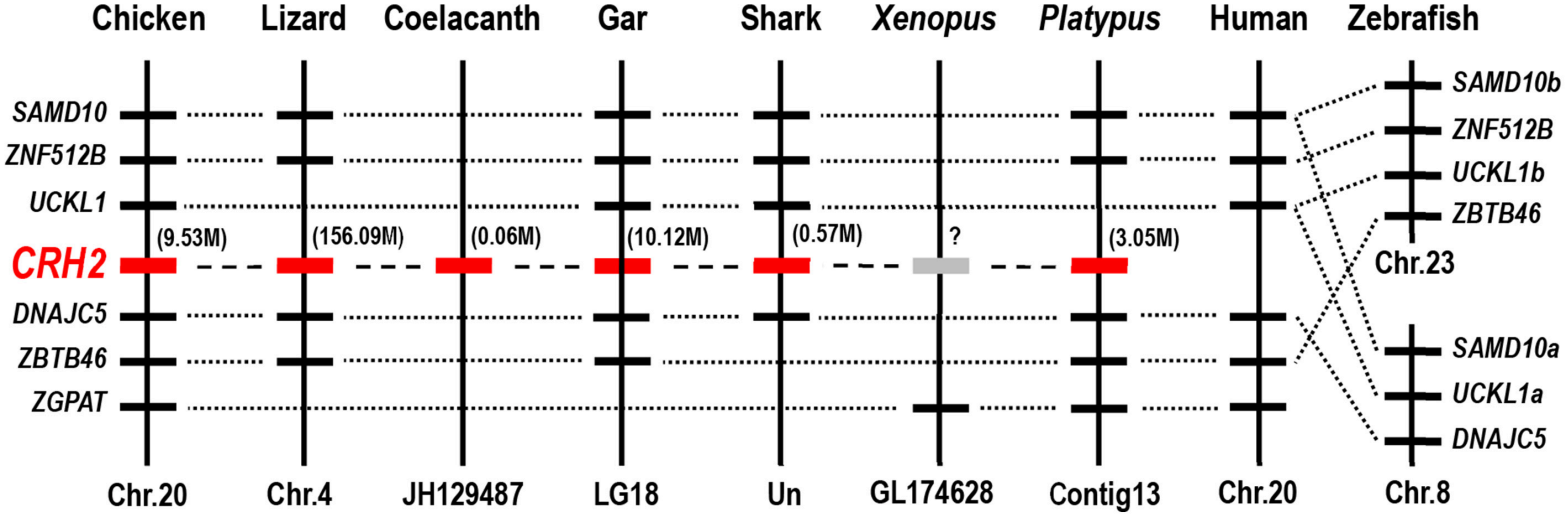
Synteny analysis showing the chromosomal location of *CRH2* in vertebrates. *CRH2* is located in a syntenic region conserved between chickens, anole lizards, coelacanth, spotted gars, sharks and platypus. Based on existing genome assemblies, *CRH2* is absent in humans and zebrafish genomes and likely lost in *Xenopus tropicalis*. Dotted lines indicate the syntenic genes; dashed lines denote genes of interest (*CRH2*); the number in bracket (M) indicate the location of *CRH2* gene in corresponding chromosome or scaffold.

To confirm that the newly cloned *cCRH2* is orthologous to *CRH2* gene in other species, synteny analysis was carried out by searching its conserved neighboring genes in genomes of several species. As expected, *CRH2* is located in a syntenic region conserved in almost all species examined, including chicken, lizard, coelacanth, spotted gar, elephant shark and platypus, suggesting the newly cloned gene is an ortholog of *CRH2*. In addition, our synteny analysis also suggested that *CRH2* is absent in humans, *Xenopus tropicalis* and zebrafish (Figure 3), as previously reported (5).

### Potency of cCRH2 in Activating Chicken CRHR1 and CRHR2

**Figure 4:**
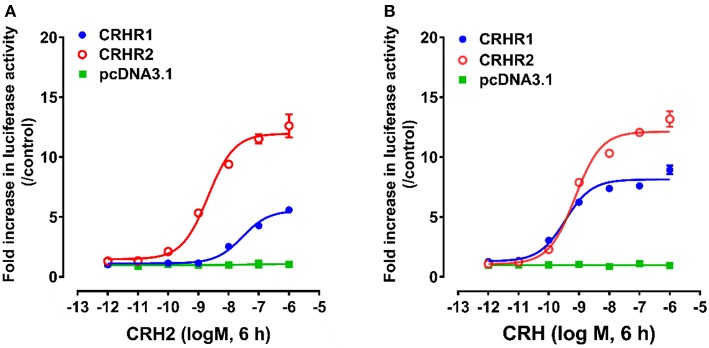
Effects of cCRH2 **(A)** or cCRH **(B)** in activating chicken CRHR1 and CRHR2 expressed in CHO cells, as monitored by the pGL3-CRE-luciferase reporter system. cCRH2 and cCRH treatment did not stimulate luciferase activity of control CHO cells co-transfected with the empty pcDNA3.1(+) vector and a pGL3-CRE-luciferase reporter construct. Each data point represents mean ±SEM of three replicates (*N* = 3).

Using pGL3-CRE-Luciferase reporter system, we further examined whether like cCRH, cCRH2 could activate the two CRH receptor subtypes (cCRHR1 and cCRHR2) transiently expressed in CHO cells (35). As shown in Figure 4, cCRH2 treatment (10^−12^-10^−6^M, 6 h) could dose-dependently stimulate luciferase activities in cCRHR1- or cCRHR2-expressing CHO cells, suggesting that both CRH receptors could be activated by cCRH2. Interestingly, cCRH2 is more potent in activating cCRHR2 (EC_50_: 1.86 nM) than cCRHR1 (EC_50_: 28.62 nM), indicating that cCRH2 is likely more selective for cCRHR2. By comparison, cCRH activates cCRHR1 and cCRHR2 with similar potency (EC_50_ values are 0.30 nM for cCRHR1 and 0.83 nM for cCRHR2), suggesting it is a common ligand for both receptors.

CHO cells co-transfected with empty pcDNA3.1(+) vector and pGL3-CRE-luciferase reporter construct were used as an internal control in parallel, and peptide treatment did not change the luciferase activity, elucidating the specific effect of peptides on receptor activation.

### Tissue Expression of CRH2 in Adult Chickens

**Figure 5:**
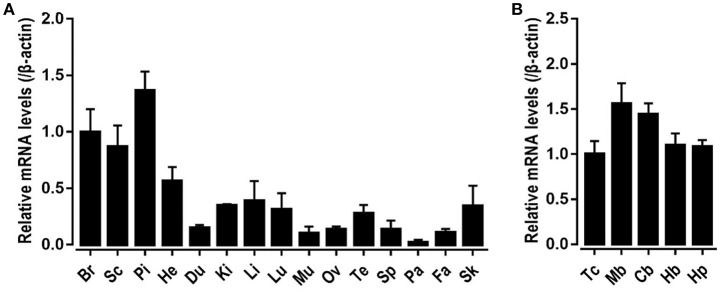
Tissue distribution of *CRH2* in adult chickens. **(A)** qPCR detection of *CRH2* expression in various chicken tissues, including the whole brain (Br), spinal cord (Sc), anterior pituitary (Pi), heart (He), duodenum (Du), kidney (Ki), liver (Li), lung (Lu), muscle (Mu), ovary (Ov), testes (Te), spleen (Sp), pancreas (Pa), fat (Fa), and skin (Sk). **(B)** qPCR assay of c*CRH2* expression in adult chicken brain regions including the telencephalon (Tc), midbrain (Mb), cerebellum (Cb), hindbrain (Hb), and hypothalamus (Hp). The mRNA levels of *CRH2* were normalized to that of β*-actin* and expressed as the fold difference compared with that of the whole brain (Br) or telencephalon (Tc). Each data point represents the mean SEM of 4 adult chickens (*N* = 4).

To explore the physiological role of *CRH2* in chickens, qRT-PCR was used to measure its mRNA expression in various adult tissues including the whole brain, spinal cord, anterior pituitary, heart, duodenum, kidneys, liver, lung, muscle, ovary, testes, spleen, pancreas, fat, and skin. As shown in Figure 5, c*CRH2* was detected to be widely expressed in all tissues examined, with abundance in the whole brain, spinal cord and anterior pituitary.

The high mRNA level of *cCRH2* in chicken brain led us to further examine its expression in various brain regions including the telencephalon, midbrain, cerebellum, hindbrain, and hypothalamus. *cCRH2* was found to be ubiquitously expressed in all brain regions examined.

### Identification of the cCRH2 Promoter Regions

**Figure 6:**
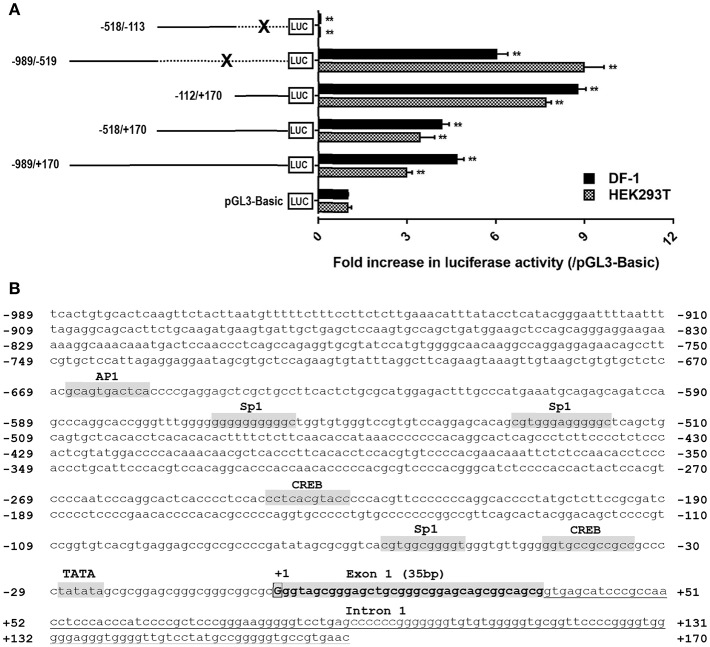
**(A)** Detection of promoter activities of the 5′-flanking region of *cCRH2* in cultured DF-1 and HEK293T cells. Various stretches of the 5′-flanking regions were cloned into pGL3-Basic vector for the generation of multiple promoter-luciferase constructs. These promoter-luciferase constructs were then co-transfected into DF-1 or HEK293T cells along with pRL-TK vector and their promoter activities were determined by the Dual-luciferase reporter assay. Each value represents the mean ± SEM of four replicates (*N* = 4). **P < 0.01 vs pGL3-Basic. Dotted line marked by an X indicates the deleted DNA fragment. **(B)** Nucleotide sequence of *CRH2* promoter region (−989/+170; Accession no. MK550695). Transcriptional start site (TSS, nucleotide “G”) determined by 5′-RACE is boxed and designated as “+1,” and the first nucleotide upstream TSS is designated as “−1.” Exon 1 (35 bp) is shaded and in bold. Tanslation start codon (ATG) is located on exon 2. The TATA box and binding sites for transcriptional factors (e.g., CREB, Sp1, AP1) were predicted using online software TFBind (http://tfbind.hgc.jp/). CREB: cAMP response element binding protein; AP1, activation protein 1; Sp1, specificity protein 1. Whether these sties are functional remains to be clarified.

Based on the transcription start site (TSS) of *cCRH2* determined by 5′-RACE in this study, we further examined whether the 5′-flanking region upstream or near exon 1 could display any promoter activity. As shown in Figure 6A, in cultured DF-1 and HEK293T cells, the construct (−989/+170Luc) exhibited an enhanced luciferase activity relative to that of the control group (pGL3-Basic vector), suggesting that the region from −989 to +170 has a promoter activity. Using the deletion approach, we noted that removal of the 5′-end (from −989 to −113) of this fragment caused an increase of luciferase activity, hinting that the core promoter region(s) of *cCRH2* is likely located within the region from −112 to +170. Interestingly, the construct (−989/−519Luc) without the fragment (−518/+170) also possessed strong promoter activity as well. This finding hinting the existence of an alternative promoter within −989/−519 region. Using the online software TFBind (http://tfbind.hgc.jp/), the putative binding sites for transcription factors such as CREB, Sp1 and AP1 were predicted within the putative promoter region from −989 to +170 (Figure 6B), further supporting that this region might be capable of driving *cCRH2* transcription.

### Effects of cCRH2 on Pituitary cTSH and cACTH Expression and Secretion

**Figure 7:**
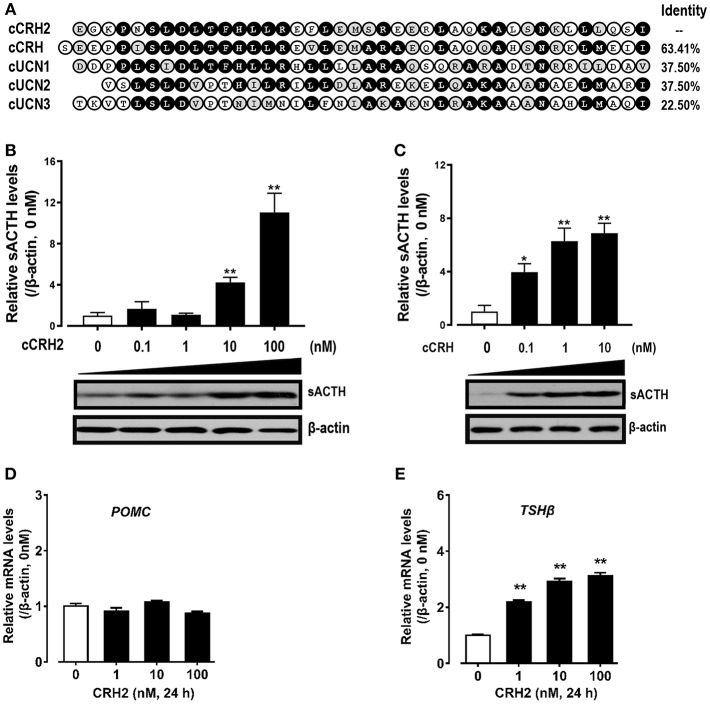
**(A)** Amino acid alignment of cCRH2 with cCRH, cUCN1, cUCN2, and UCN3. Identical amino acid residues were shaded in black, and similar ones were labeled in gray. **(B,C)**Western blot showing dose-dependent effects of cCRH2 **(B)** and cCRH **(C)** on ACTH secretion in cultured chick pituitary cells. Pituitary cells were incubated with designated dose of cCRH2 (0.1–100 nM) or cCRH (0.1–10 nM) for 6 h, then the culture medium and cell lysate were used for Western blot detection of ACTH and β-actin, respectively. The sACTH band at ~5 kDa (secretory ACTH level detected in culture medium) were semi-quantified by densitometric analysis. Their relative levels were normalized by that of β-actin in pituitary cell lysate, and then expressed as fold increase compared to control (0 nM). Each data point represents mean SEM of 3 replicates (*N* = 3). *P < 0.05, **P < 0.01, vs. control (0 nM). Representative set of Western blot is shown at the bottom of each graph; **(D,E)** qPCR assay of cCRH2 (1–100 nM) effect on pituitary *POMC* **(D)** and *TSH*β **(E)** expression. Each data point represents mean SEM of 4 replicates (*N* = 4).**P < 0.01 vs. control (0 nM).

Given that cCRH2 showed the highest similarity with cCRH (Figure 5A) and its mRNA is widely expressed in chicken tissues including the hypothalamus and anterior pituitary (Figure 4), we hypothesized that cCRH2 may function in the chicken pituitary analogous to CRH, such as stimulating TSH and ACTH expression and secretion. To elucidate this, the effect of cCRH2 on ACTH and TSHβ expression/secretion was evaluated in cultured chick pituitary cells by Western blot. As shown in Figures 7B,C, like cCRH, cCRH2 treatment could stimulate ACTH secretion into the culture medium. However, we noted that comparatively high concentration of cCRH2 (≥10 nM) was required to induce ACTH secretion significantly, while cCRH could potently stimulate ACTH secretion even at 0.1 nM. This hints that cCRH2 has a much lower potency in stimulation of ACTH secretion when compared to cCRH. Although cCRH2 could stimulate ACTH secretion, cCRH2 treatment (1–100 nM) for 24 h has no significant effect on *POMC* mRNA expression (Figure 7D).

In addition to ACTH secretion, cCRH2 could also strongly stimulate *TSH*β mRNA expression with the minimal effective dose of 1 nM (Figure 7E). Despite the high potency of CRH2 in stimulating *TSH*β expression, we failed to observe the consistent stimulatory action of CRH2 on TSH secretion from chick pituitary incubated *in vitro* in our preliminary study (*data not shown*).

## Discussion

Recently, the novel *CRH2* was reported in vertebrates except teleosts and placental mammals (5, 30), however, its expression, functionality and physiological role remain an open question. In this study, using chicken as an animal model, the full-length cDNA encoding CRH2 was cloned. Functional assays confirmed that CRH2 is bioactive and preferentially activates cCRHR2, rather than cCRHR1. qRT-PCR revealed that *CRH2* is widely expressed in chicken tissues, including the hypothalamus and pituitary. Moreover, we demonstrated that cCRH2 can potently stimulate *TSH*β expression and shows a lower potency in inducing ACTH secretion *in vitro*. To our knowledge, our study represents the first to characterize the functionality and physiological roles of CRH2 in vertebrates.

In 2011, *in silico* analysis first identified a novel *CRH* paralog, i.e., *CRH2*, from the genome sequence of elephant shark (31). This gene was later identified in other vertebrates as well, but was proposed to be lost in the teleost and placental mammal lineages. In 2015, Grone et al. predicted that *CRH2* only exists in the genomes of some birds (ducks, zebra finches, and peregrine falcons), and is likely lost in some avian lineages such as chickens and turkeys (30). However, in this study, the full-length cDNA encoding cCRH2 was cloned from chicken brain and synteny analysis revealed that it is orthologous to *CRH2* of spotted gar, elephant shark, lizard and platypus. Considering the high GC-rich content of *cCRH2* cDNA (~72%) and the difficulty in sequencing, it is not surprising why previous studies have failed to identify *CRH2* in some avian lineages, possibly due to gaps in the genome assemblies (5, 30). Following this line of argument, we infer that *CRH2* may exist in all avian species, nevertheless, this assumption warrants further elucidation. However, concurring with previous report that *CRH2* has undergone gene loss in teleosts and placental mammals (5, 22), we could not find this gene in zebrafish and humans. In addition, we failed to identify *CRH2* in genome database of *Xenopus tropicalis*, therefore it is difficult to conclude whether it exists in amphibians.

Like other CRH-family members, cCRH2 shares the same exon-intron organization and its precursor is also encoded by the second exon (5, 37). In spite of the limited sequence identity shared between the precursors of chicken CRH family members, amino acid sequence alignment showed CRH2 shares the highest identity with CRH in term of precursor sequence (31.14%) among chicken CRH family members (Figure 2). Moreover, the mature peptide of cCRH2 shows the highest sequence identity (63.41%) to that of cCRH. These results indicate that *CRH2* and *CRH* are the closest in term of phylogenetic origin among the 5 identified CRH family members, supporting the notion that *CRH2* was possibly derived from the second round of vertebrate genome (2R) duplication, which occurred after the emergence of the joint CRH/UTS1 ancestor in the first round of vertebrate whole-genome duplication (1R) (5).

In this study, we also found that like cCRH, cCRH2 can activate both CRH receptors (CRHR1 and CRHR2) and stimulate the intracellular cAMP/PKA signaling pathway. However, we found that CRH2 preferentially activates cCRHR2. This differs from CRH which has equipotent activation of both receptors (27). Our data, for the first time, proved that CRH2 has a biological activity similar, but not identical to, that of CRH in vertebrates.

To investigate the physiological roles of *CRH2* in chickens, we first examined its spatial expression by qPCR. c*CRH2* is widely expressed in various chicken tissues. This finding is different from the previous report in spotted gars, in which *CRH2* mRNA is restricted in the isthmic region of the hindbrain, as detected by *in situ* hybridization (30). Grone et al., speculated that CRH2 might be involved in a more specialized function, i.e., feeding or automatic regulation, based on its limited expression profile. However, our finding suggests that cCRH2 may play diverse roles in the CNS and peripheral tissues of chickens. Similar to the spatial expression profile of *CRH* in mammals and *Xenopus laevis* (38), c*CRH2* mRNA was detected in all brain regions and the anterior pituitary, further supporting the possibility that cCRH2 may play roles, similar to those of cCRH, such as inhibiting food intake and activating the HPA axis in chickens.

Based on the putative transcriptional start site of *cCRH2* determined by 5′-RACE PCR in our study, the promoter activity of its 5′-flanking regions (−989/+170) located near exon 1 was also analyzed. The two regions of −989/−519 and −112/+170 possess strong promoter activities, indicating they are likely important in regulating *CRH2* transcription. Interestingly, based on our promoter deletion results, we found that the −518/−113 region contributes to a reduction in promoter activities, hinting the possible presence of silencers in this region (39). Considering the ubiquitous *CRH2* expression in adult chicken tissues, we propose that *CRH2* transcription might be driven by multiple promoters located in −989/+170 region. Nevertheless, further studies are required to substantiate this hypothesis. Interestingly, like vertebrate *CRH* (40–42), *cCRH2* proximal promoter region also contains putative binding sites for transcriptional factors, e.g., CREB and AP1 (Figure 7B), which are reported to be involved in repressing or promoting *CRH* expression (40–42), however, it remains unclear whether like *CRH*, c*CRH2* transcription could also be controlled by these predicted *cis-*regulatory elements.

**Figure 8:**
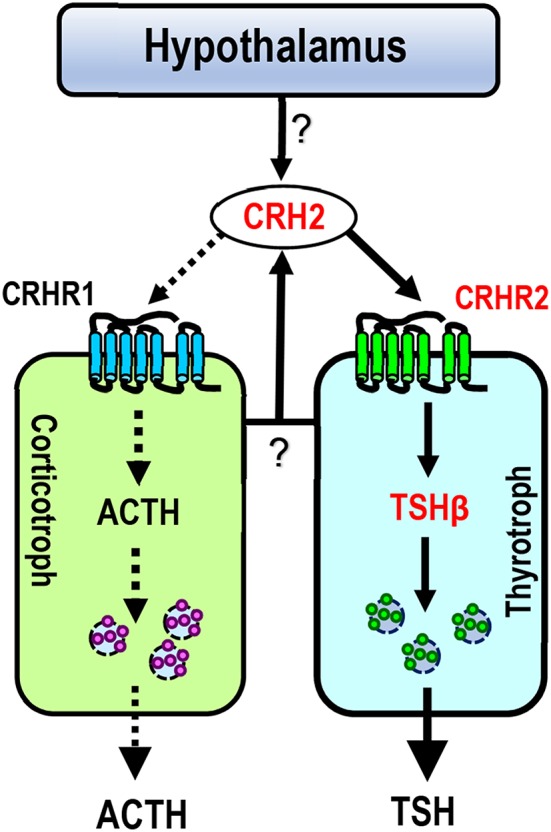
Proposed model for CRH2 action on chicken anterior pituitary. Like CRH, CRH2 (produced in the pituitary or hypothalamus) can potently stimulate TSH expression via the activation of CRHR2 expressed in thyrotrophs (marked by solid lines), suggesting that cCRH2 may play an important role in the hypothalamus-pituitary-thyroid axis (HPT axis). Unlike CRH, CRH2 has a low potency (≥10 nM) in stimulating ACTH secretion (marked by dotted lines) possibly via activating CRHR1 expressed in corticotrophs. In this model, CRH2 is expressed in the hypothalamus-pituitary axis, however, it remains to be clarified which type(s) of pituitary cell secrete CRH2, or whether hypothalamic CRH2 can directly control TSH/ACTH expression and/or secretion (marked by question marks).

The relatively high mRNA level of c*CRH2* detected in the chicken hypothalamus-pituitary axis suggests that cCRH2 may possess some “CRH-like actions” in the anterior pituitary, such as stimulating pituitary ACTH and TSH expression or secretion (43). Our study confirmed that cCRH can potently stimulate ACTH release in cultured chick pituitary cells, which is identical to previous reports in chickens, mammals and tilapia (4, 8, 43, 44). However, we found that cCRH2 is, at least, 10-fold less potent than cCRH in inducing ACTH secretion in cultured pituitary cells (Figure 7). In chickens, it is reported that CRHR1, but not CRHR2, could mediate the roles of CRH in stimulating ACTH secretion (45, 46). Since CRH2 could activate CRHR1 only at high concentrations, it is likely that high doses of cCRH2 are required to activate CRHR1 expressed in corticotrophs, which subsequently induce ACTH release in the chicken pituitary (Figure 8). Besides stimulating of ACTH secretion, CRHR1 signaling is reported to be capable of stimulating pituitary CART secretion and expression (27, 47). Considering the low potency of CRH2 in activating CRHR1 (Figure 4), it may also imply that CRH2 may play a limited role in the regulation of chicken pituitary CART release and synthesis.

CRH is believed to be responsible for stimulating pituitary TSH secretion and synthesis in some non-mammalian vertebrates including chickens (43, 48–50), and this action was proved to be mediated by CRHR2 (16, 45, 48, 51). The relatively high potency of cCRH2 in activating cCRHR2 led us to examine whether like cCRH, cCRH2 can stimulate TSH expression in the chicken pituitary (Figure 8). In agreement with this hypothesis, we proved that CRH2 could potently stimulate *TSH*β expression. Our finding raises a new concept that apart from CRH, TRH and GCG-like peptide (GCGL) [which are reported to stimulate chicken pituitary TSH expression/secretion (52–54)], CRH2 derived from the pituitary or hypothalamus is likely another important player in the regulation of the hypothalamus-pituitary-thyroid (HPT) axis activity in chickens (Figure 8).

In summary, the novel *CRH2* gene was identified in chickens. Functional assays elucidated that cCRH2 is 15-fold more potent in activating cCRHR2 than cCRHR1. qPCR revealed that *cCRH2* is widely expressed in chicken tissues including the hypothalamus and anterior pituitary. Moreover, CRH2 can potently stimulate pituitary *TSH*β mRNA expression and induce ACTH secretion only at high concentration (≥10 nM). Evidence presented here, for the first time, establish a novel concept that CRH2 is biologically active and regulates pituitary functions in vertebrates.

## Data Availability

The datasets generated for this study can be found in the Full-length cDNA of cCRH2 (NCBI genbank Accession No. KU887752).

## Author Contributions

GB, JF, MY, CL, YL, JiL, FM, XD, JZ, and JuL conducted the experiments. All authors joined the analysis and interpretation of data. GB, XZ, and YW designed and drafted the manuscript. All authors read and approved the final manuscript.

### Conflict of Interest Statement

The authors declare that the research was conducted in the absence of any commercial or financial relationships that could be construed as a potential conflict of interest.
